# Upper critical field reaches 90 tesla near the Mott transition in fulleride superconductors

**DOI:** 10.1038/ncomms14467

**Published:** 2017-02-17

**Authors:** Y. Kasahara, Y. Takeuchi, R. H. Zadik, Y. Takabayashi, R. H. Colman, R. D. McDonald, M. J. Rosseinsky, K. Prassides, Y. Iwasa

**Affiliations:** 1 Department of Physics, Kyoto University, Kyoto 606-8502, Japan; 2 Quantum-Phase Electronics Center (QPEC) and Department of Applied Physics, University of Tokyo, Tokyo 113-8656, Japan; 3 Department of Chemistry, Durham University, Durham DH1 3LE, UK; 4 WPI—Advanced Institute for Materials Research, Tohoku University, Sendai 980-8577, Japan; 5 NHMFL, Los Alamos National Laboratory, Los Alamos, New Mexico 87545, USA; 6 Department of Chemistry, University of Liverpool, Liverpool L69 7ZD, UK; 7 Japan Science and Technology Agency (JST), ERATO Isobe Degenerate π-Integration Project, Tohoku University, Sendai 980-8577, Japan; 8 RIKEN Center for Emergent Matter Science (CEMS), Wako, Saitama 351-0198, Japan

## Abstract

Controlled access to the border of the Mott insulating state by variation of control parameters offers exotic electronic states such as anomalous and possibly high-transition-temperature (*T*_c_) superconductivity. The alkali-doped fullerides show a transition from a Mott insulator to a superconductor for the first time in three-dimensional materials, but the impact of dimensionality and electron correlation on superconducting properties has remained unclear. Here we show that, near the Mott insulating phase, the upper critical field *H*_c2_ of the fulleride superconductors reaches values as high as ∼90 T—the highest among cubic crystals. This is accompanied by a crossover from weak- to strong-coupling superconductivity and appears upon entering the metallic state with the dynamical Jahn–Teller effect as the Mott transition is approached. These results suggest that the cooperative interplay between molecular electronic structure and strong electron correlations plays a key role in realizing robust superconductivity with high-*T*_c_ and high-*H*_c2_.

## Abstract (web summary)

Alkali-doped fullerides are superconductors but the impact of dimensionality and electron correlation remains unclear. Here, Kasahara *et al*. report an upper critical field about 90 T, suggesting cooperative interplay between molecular electronic structure and strong electron correlations.

The interplay between superconductivity and electron correlations is one of the central issues in condensed matter physics. Superconducting (SC) materials based on Mott insulators, such as two-dimensional (2D) cuprates^1^ and organic charge-transfer salts^2^, are model platforms that have been extensively studied thus far. A dome-like dependence of the SC transition temperature *T*_c_ as a function of tuning parameters, such as carrier doping and pressure, has been discussed as a fingerprint of unconventional superconductivity^3^. Recent physical and chemical pressure studies of Cs_3_C_60_ have revealed that the family of cubic fullerides A_3_C_60_ (A: alkali metal), where superconductivity emerges from the Mott insulating state driven by dynamical intramolecular Jahn–Teller (JT) distortions and strong Coulomb repulsion, is a new example of superconductors that show a dome-like SC phase diagram as a function of unit-cell volume *V* (refs 4, 5, 6, 7, 8, 9). This suggests the importance of strong electron correlation to SC mechanisms^10^ and the need for further treatment beyond conventional framework of theory^11^. Recent study has revealed a crossover in the normal state from the conventional Fermi liquid to a nontrivial metallic state where JT distortions persist (JT metal)^9^,^12^. There, localized electrons coexist with itinerant electrons microscopically and heterogeneously.

The dependence of the upper critical field *H*_c2_ on *T*_c_ is relevant to the understanding of the dome-like SC phase because *H*_c2_ is determined by the coherence length (the size of the Cooper pair) as well as the strength of the pairing potential. Therefore, *H*_c2_ is also important to understand the underlying mechanism of the superconductivity. However, for the fullerides, *H*_c2_ as a function of *V* has not as yet been determined due to the very large *H*_c2_ and the need for high pressure to access superconductivity in Cs_3_C_60_.

Here we report measurements of *H*_c2_ using a pulsed magnetic field in Rb_*x*_Cs_3−*x*_C_60_, where superconductivity appears near the Mott transition even at ambient pressure^9^. In proximity to the Mott transition, *H*_c2_ is enhanced up to ∼90 T, which is the highest among cubic superconductors. We uncovered that *H*_c2_ and the pairing strength increase concomitantly with increasing lattice volume near the Mott transition, suggesting that molecular characteristics as well as electron correlations play important roles for realizing superconductivity with high-*T*_c_ and high-*H*_c2_ in molecular materials.

## Results

### Temperature dependence of upper critical field

**Figure 1:**
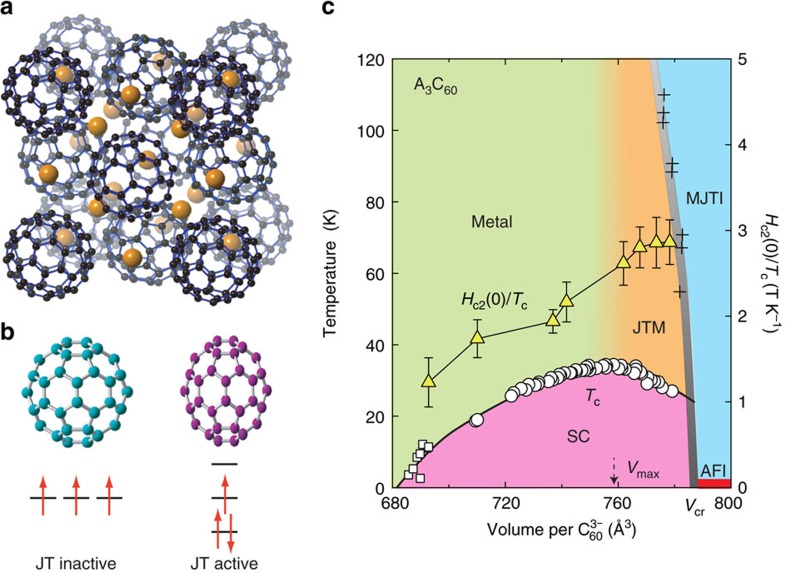
Crystal structure and electronic phase diagram of fcc fullerides. (**a**) Crystal structure of fcc A_3_C_60_. Orange and black spheres represent A and C atoms, respectively. The 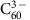 anions adopt two orientations related by 90^°^ rotation about the [100] axis. Only one is shown at each site. (**b**) Schematic structures of 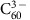 anions and molecular *t*_1*u*_ orbitals. At low *V*, 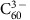 anions are isotropic, and *t*_1*u*_ orbitals are triply degenerate. At large *V*, dynamical JT distortions give rise to threefold splitting of the *t*_1*u*_ orbitals. (**c**) Electronic phase diagram of cubic fullerides. Squares and circles are the superconducting (SC) transition temperature *T*_c_ for f.c.c. 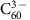 anion packings with *Pa*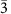 symmetry and *Fm*
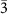
*m* symmetry, respectively. In fcc-Cs_3_C_60_ at ambient pressure, an electron-correlation-driven insulating state (Mott-Jahn-Teller insulator, MJTI) appears, which is accompanied by an intramolecular dynamical Jahn–Teller (JT) effect distorting the 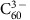 anions and stabilizing the low-spin (*S*=1/2) states that give rise to an antiferromagnetic insulating (AFI) state at low temperatures. In the metallic regime, gradient shading from green to orange schematically illustrates a crossover from the conventional metal to unusual metallic state where JT distortions persist, which we define as the JT metal (JTM) state. The grey line represents the MJTI-to-JTM crossover line, where the crossover temperatures (crosses) were obtained from X-ray powder diffraction, nuclear magnetic resonance spectroscopy, and infrared spectroscopy^9^. The ratio of upper critical field at *T*=0 and *T*_c_, *H*_c2_(0)/*T*_c_ (yellow triangles), shows an enhancement in the JTM regime. Error bars represent the s.d. in the values of *H*_c2_(0) estimated from the least-squares fits of equation (1) to *H*_c2_(T) data.

**Figure 2:**
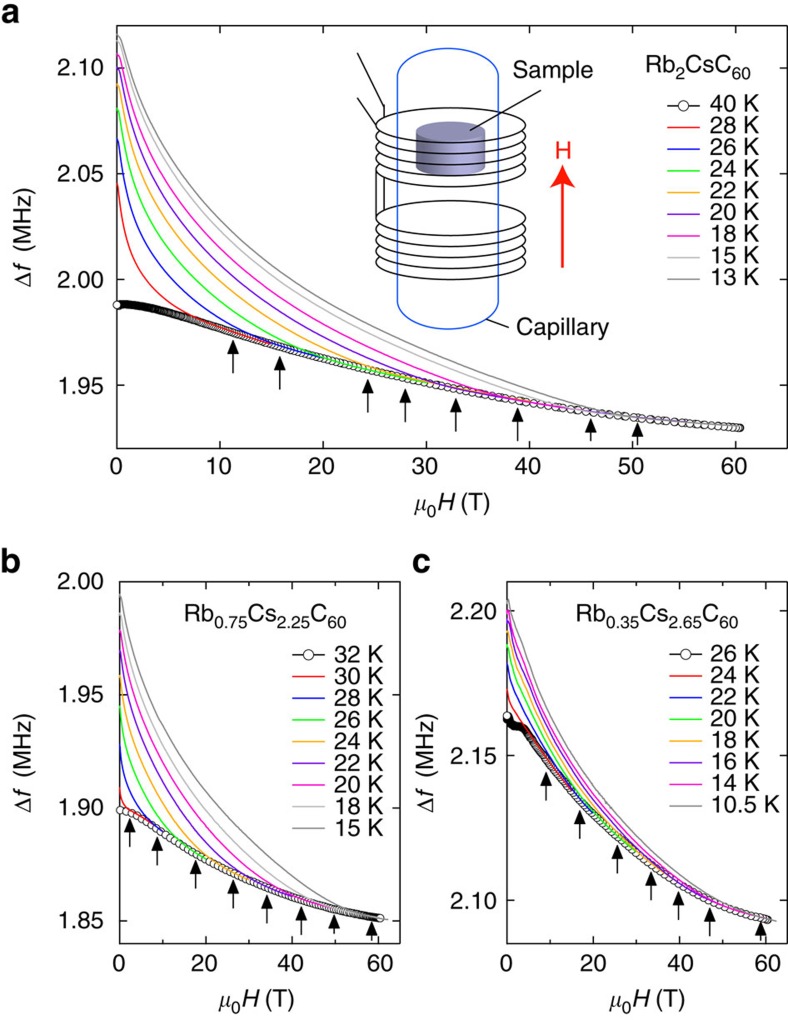
Determination of upper critical fields. Frequency shift (Δ*f*) as a function of magnetic field for Rb_*x*_Cs_3−*x*_C_60_ with (**a**) *x*=2, (**b**) 0.75, and (**c**) 0.35 at selected temperatures. Open circles are Δ*f* taken at *T*>*T*_c_ as a normal-state background signal. The arrows indicate *H*_c2_(T) determined from the point deviating from the background signal. Inset in **a** shows a schematic of the experimental set-up. The sample in a capillary was inserted in one coil of the pair wound clockwise and anti-clockwise to compensate induced voltages that are generated during the field pulse.

**Figure 3:**
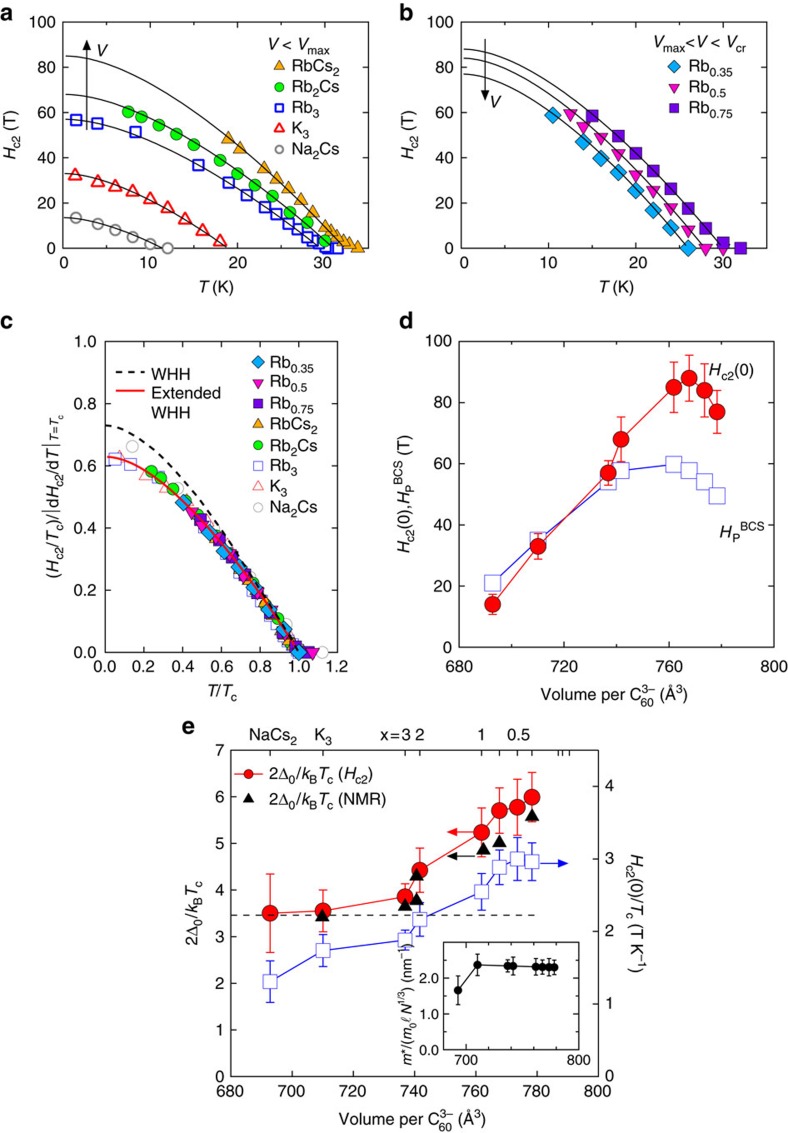
Upper critical field in fullerene superconductors. Temperature dependence of the upper critical field for (**a**) *V*≦*V*_max_ and for (**b**) *V*_max_<*V*<*V*_cr_. The solid lines represent fits using equation (1). (**c**) Normalized *H*_c2_, 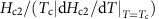, as a function of normalized temperature *T*/*T*_c_. The solid and dashed lines represent calculated *H*_c2_(*T*) using the extended WHH formula (equation (1)) with *α*=1.5 and *λ*_so_=4.4 and the conventional WHH formula in the dirty limit, respectively. (**d**) *H*_c2_(0), obtained from fits using equation (1), are plotted as a function of volume per 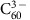 anions. Error bars represent s.d. of the fit to *H*_*c*2_(*T*) curves. Conventional BCS values of the Pauli limiting field 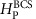 are also shown. (**e**) Evolution of 2Δ_0_/*k*_B_*T*_c_ and *H*_c2_(0)/*T*_c_ with approaching to the Mott transition. Error bars on 2Δ_0_/*k*_B_*T*_c_ and *H*_c2_(0)/*T*_c_ are calculated from the s.d. in the values of *H*_c2_(0) estimated from the least-squares fits of equation (1) to *H*_c2_(T) data. 2Δ_0_/*k*_B_*T*_c_ obtained from NMR measurements is taken from ref. 9. Inset shows *V* dependence of 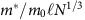 derived using 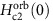, Δ_0_, and *V*. Error bars are calculated from the s.d. in the values of *H*_c2_(0) estimated from the least-squares fits of equation (1) to *H*_c2_(T) data..

*H*_c2_ of the fulleride superconductors (Fig. 1a) Na_2_CsC_60_, K_3_C_60_, and Rb_*x*_Cs_3−*x*_C_60_ (0<*x*≦3), has been measured by a radiofrequency technique in pulsed magnetic fields^13^ up to 62 T (see Methods). In Rb_*x*_Cs_3−*x*_C_60_ with *x*≦1, the dynamical JT distortions (Fig. 1b) persist down to low temperature and coexist with the metallic state, and superconductivity emerges from this JT metal state (*V*_max_<*V*<*V*_cr_, in Fig. 1c). Figure 2 shows temperature (*T*) variations of frequency shift Δ*f* as a function of the magnetic field *H* for Rb_*x*_Cs_3−*x*_C_60_ (*x*=2, 0.75, and 0.35) (see also Supplementary Fig. 1). The *T* dependence of *H*_c2_, *H*_c2_(*T*), was determined as a point at which Δ*f* intercepts the normal-state background (arrows in Fig. 2). *H*_c2_(*T*) curves for A_3_C_60_ are plotted in Fig. 3a,b for *V*≦*V*_max_ and *V*_max_<*V*<*V*_cr_ in the proximity of the Mott transition, respectively. *H*_c2_(*T*) increases linearly with decreasing *T* near *T*_c_ and has a tendency to saturate at low temperatures. No obvious upturn of *H*_c2_(*T*) is found in any of the samples measured, implying that *H*_c2_(*T*) can be understood within a simple single-band picture despite the multiband nature of the triply degenerate *t*_1u_ orbitals of 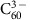 anions, in contrast to MgB_2_ and iron pnictides where multiband and multigap behaviour with upturn or quasilinear *T* dependence down to *T*∼0 is commonly observed.

### Volume dependence

In spin-singlet superconductors, *H*_c2_ is determined by two distinct effects, i.e., the orbital and the Pauli paramagnetic effect. The orbital limit and Pauli limit are given by 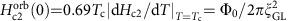 and 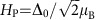, respectively (Φ_0_, *ξ*_GL_, Δ_0_, and *μ*_B_ are the flux quantum, Ginzburg–Landau (GL) coherence length, superconducting gap and Bohr magneton, respectively)^14^,^15^. In a weak-coupling BCS superconductor, the Pauli limit is 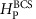
[T]=1.84*T*_c_[K]. A simple estimation from 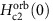 gives *ξ*_GL_=1.8–4.6 nm (Supplementary Table 1), which is comparable to the lattice constant. It should be noted that the fulleride superconductors are in the dirty limit, ℓ≲*ξ*_0_ (ℓ and *ξ*_0_ are the mean free path and Pippard coherence length, respectively), as demonstrated by transport and optical measurements^16^,^17^. The orientational disorder of the 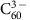 anions can account for the short ℓ, which is comparable to the intermolecular separation. The relation 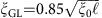 in the dirty limit, where *ξ*_0_=*ħv*_F_/*π*Δ_0_ and 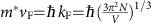 (*v*_F_, *m*, *k*_F_, and *N* are the Fermi velocity, effective mass, Fermi momentum, and number of electrons per C_60_, respectively) for the parabolic band approximation yield 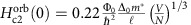. In the extreme cases (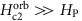 or 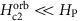), *H*_c2_(0) is determined solely by *H*_c2_^orb^ or *H*_P_. However, when these two quantities are comparable, *H*_c2_(*T*) can be described by the extended WHH formula^14^, which considers both the orbital and Pauli paramagnetic effects as well as spin–orbit scattering,


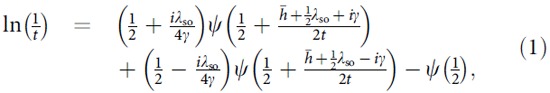


where *t*=*T*/*T*_c_, 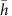
=0.281*H*_c2_(*T*)/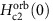, 
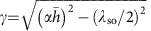
, 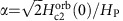, 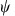 is the digamma function, and *λ*_so_ is the spin–orbit scattering constant. With fixed 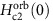, finite *α* reduces *H*_c2_(0), but it recovers toward the original value with increasing *λ*_so_, since spin–orbit scattering suppresses the Pauli paramagnetic effect.


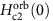
 was estimated from the initial slope of *H*_c2_(*T*) since the Pauli paramagnetic effect is not relevant near *T*_c_ (Supplementary Note 1; Supplementary Fig. 2; and Supplementary Table 1). Then, *H*_c2_(*T*) curves were fitted with *H*_P_ and *λ*_so_ as fitting parameters. As shown by the solid lines in Fig. 3a,b, *H*_c2_(*T*) curves are well described by equation (1). Figure 3c shows *H*_c2_(*T*) normalized by 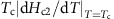 as a function of *T*/*T*_c_. The normalized *H*_c2_(*T*) curves collapse into a single curve except for Na_2_CsC_60_, implying that the parameters *α* and *λ*_so_ are unchanged in a wide *V* region of the phase diagram, resulting in (*α*, *λ*_so_)=(1.5, 4.4). Figure 3d displays the evolution of *H*_c2_(0) as a function of *V*, together with 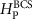. *H*_c2_(0) reaches as high as 88 T in Rb_*x*_Cs_3−*x*_C_60_ with *x*≦1 (*V*≧*V*_max_) very close to the Mott transition. Moreover, *H*_c2_(0) is clearly larger than 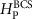 at *V*>*V*_max_, and the difference between *H*_c2_(0) and 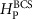 becomes pronounced with increasing *V*, although *T*_c_ is almost unchanged near the Mott transition.

## Discussion

*H*_c2_(0) values reaching ∼90 T are remarkably high for 3D materials. Typical examples of 3D superconductors are cubic Nb_3_Sn (*H*_c2_(0)=30 T, *T*_c_=18 K), which is well known as a material for a SC magnet^18^, and Ba_1−*x*_K_*x*_BiO_3_ (*H*_c2_(0)=32 T, *T*_c_=28 K)^19^. MgB_2_ exhibits strong anisotropy (*H*_c2_(0)=49 T and 34 T parallel to the *ab* plane and *c* axis, respectively, *T*_c_=39 K)^18^ due to its anisotropic electronic structure. *H*_c2_(0) of the fullerides is even higher than that of recently discovered H_3_S superconductors with likely a cubic structure (*H*_c2_(0)≈70 T, *T*_c_=203 K)^20^ despite its much higher *T*_c_. In 2D systems under in-plane applied fields, the orbital effect is quenched and higher *H*_c2_ can be expected. Very large *H*_c2_ compared with low *T*_c_ has been demonstrated in ion-gated MoS_2_ (*H*_c2_(0)=52 T, *T*_c_=9.7 K)^21^,^22^ and monolayer NbSe_2_ (*H*_c2_(0)=32 T, *T*_c_=3.0 K)^23^. In the bulk materials, the in-plane *H*_c2_ of the cuprates is exceptionally high at above 100 T. However, *H*_c2_ is no longer a thermodynamic transition line, but a crossover line due to thermal fluctuations. Contrastingly, *H*_c2_ in pnictides with *T*_c_≃30 K is as large as that of fullerides^24^. Therefore, our results highlight the uniquely high *H*_c2_ measured in the fulleride superconductors that are cubic, and thus, 3D.

To understand the underlying mechanisms for the evolution of *H*_c2_(0), we estimated unknown parameters that determine *H*_P_ and 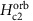 (Supplementary Fig. 1), that is, Δ_0_ and the product of parameters in the normal state 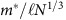. Δ_0_ can be directly estimated from *H*_P_. In Fig. 3e, the *V* dependences of 2Δ_0_/*k*_B_*T*_c_, which is related to the strength of the pairing interaction, and 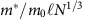 (*m*_0_ is the bare electron mass) are shown. At low *V*, 2Δ_0_/*k*_B_*T*_c_ is comparable to the BCS weak-coupling limit value of 3.52. In contrast with the dome-shaped *T*_c_, 2Δ_0_/*k*_B_*T*_c_ continuously increases with increasing *V* and reaches values as large as 6, indicating a crossover from weak- to strong-coupling superconductivity on approaching the Mott transition. This is in good agreement with the previous nuclear magnetic resonance results for Rb_*x*_Cs_3−*x*_C_60_ at ambient pressure^9^ and for both fcc- and A15-Cs_3_C_60_ under pressure^25^,^26^, implying universal behaviour in the fullerides. On the other hand, 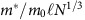 is almost constant, indicating that both *H*_P_/*T*_c_ and 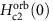/*T*_*c*_ are solely proportional to 2Δ_0_/*k*_B_*T*_*c*_. These results lead to the conclusion that the enhancement of *H*_c2_(0) is dominated by the strong-coupling effect developing near the Mott transition.

**Figure 4:**
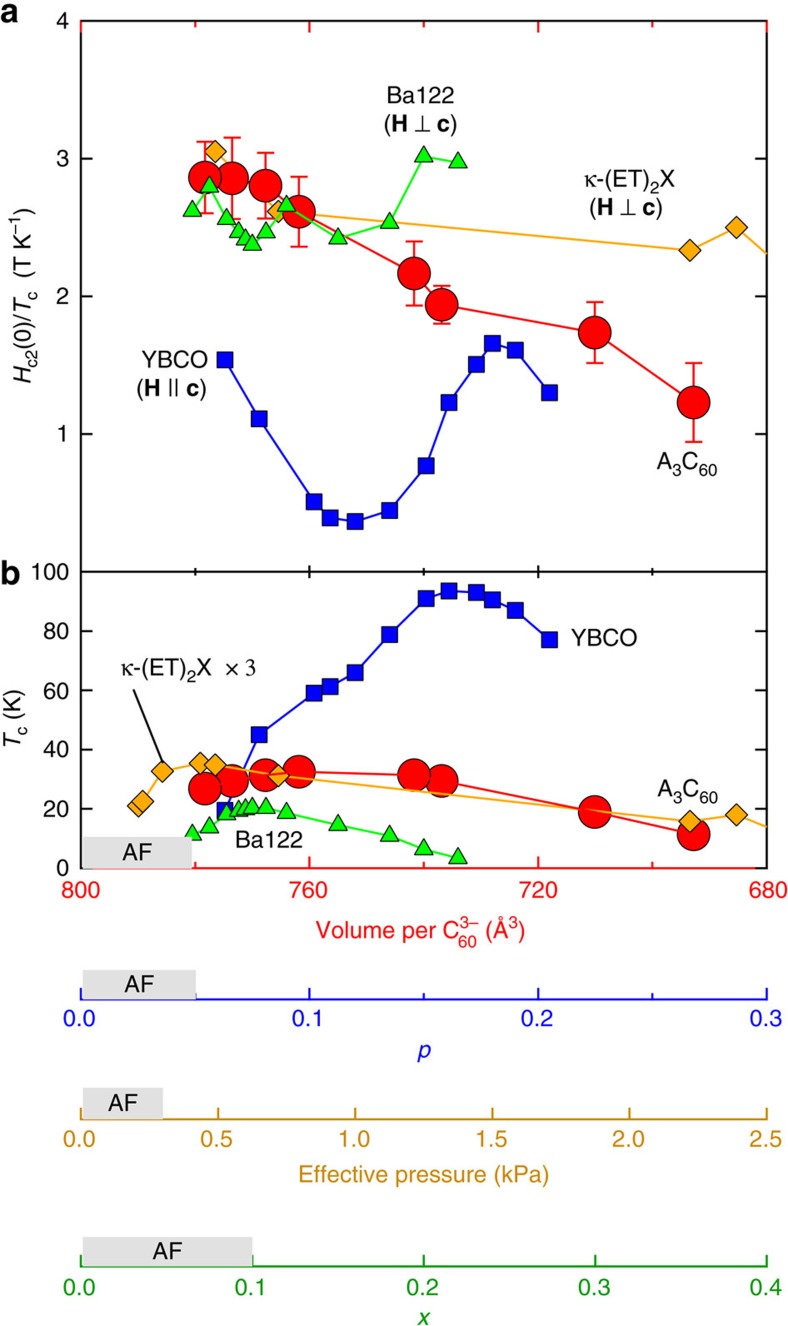
Comparison of upper critical field and *T*_c_ as a function of control parameters in high-*T*_c_ superconductors. Variations of (**a**) *H*_c2_(0)/*T*_c_ and (**b**) *T*_c_ in high-*T*_c_ superconductors, including fullerides A_3_C_60_, cuprates YBa_2_Cu_3_O_*y*_ (YBCO) (ref. 27), iron-pnictides BaFe_2−*x*_Ni_*x*_As_2_ (ref. 24), and organic charge-transfer salts κ-(BEDT-TTF)_2_X (X=Cu(NCS)_2_ and Cu[N(CN)_2_]Br) (refs 28, 29, 30), plotted as a function of control parameters, i.e., lattice volume per 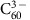 (*V*), hole concentration (*p*), Ni content (*x*), and effective pressure measured from κ-(BEDT-TTF)_2_Cu[N(CN)_2_]Cl (ref. 31). Error bars on *H*_c2_(0)/*T*_c_ for fullerides represent the s.d. in the values of *H*_c2_(0) estimated from the least-squares fits of equation (1) to *H*_c2_(T) data.

We here recall *H*_c2_(0) of other families of high-*T*_c_ or strongly correlated superconductors, i.e., cuprates, organic κ-(ET)_2_X, and pnictides^24^,^27^,^28^,^29^,^30^,^31^, having a dome-like SC phase and a proximate antiferromagnetic phase. In Fig. 4, *H*_c2_(0)/*T*_c_ is displayed as a function of the relevant tuning parameter for each materials family. We show *H*_c2_(0) for the in-plane field (**H** ⊥ **c**), where the Pauli paramagnetic effect is dominating, in κ-(ET)_2_X and pnictides but show *H*_c2_(0) for the out-of-plane field (**H** || **c**) in cuprates since there are no reliable estimates of *H*_c2_(0) for **H** ⊥ **c**. A remarkable feature of the fullerides is that *H*_c2_(0)/*T*_c_ appears to be strongly enhanced at *x*≤1, where the JT metal phase emerges (Fig. 1c), with retaining nearly optimal *T*_c_ and *H*_c2_(0) values near the Mott transition. This is in marked contrast to the pnictides and cuprates. In the pnictides, *H*_c2_(0)/*T*_c_ is almost constant across the SC dome. This is ascribed to the variation of Δ_0_, which linearly scales with *T*_c_ (ref. 32), implying constant coupling strength. Moreover, in pnictides, *T*_c_ and *H*_c2_(0) are strongly reduced upon decreasing doping, associated with the appearance of the antiferromagnetic phase. Non-monotonic behaviour in cuprates appears with mass enhancement near *p*=0.08 and 0.18, which originates from phase competition between superconductivity and Fermi-surface reconstruction or charge-density-wave order^27^. This is distinct from the continuous evolution of 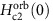 in the fullerides (Supplementary Fig. 2), suggesting the absence of such competing states. In κ-(ET)_2_X, there is no competing phase near the Mott transition and the molecular degrees of freedom are not relevant to the superconductivity in contrast to the fullerides. Moreover, the SC pairing is most likely mediated by purely electronic interaction, in contrast to the fullerides, where there is considerable controversy because of comparable energy scales in the electron–phonon and electron-electron interactions^33^,^34^. κ-(ET)_2_X shows qualitatively similar behaviour with the strong-coupling effects near the antiferromagnetic phase^35^. However, the enhancement of *H*_c2_(0)/*T*_c_ is much weaker than that in the fullerides. Therefore, the steep enhancement of *H*_c2_(0)/*T*_c_ and 2Δ_0_/*k*_B_*T*_c_ upon entering the JT metal phase cannot be explained solely by the electron correlation effect, highlighting the uniqueness of fullerides among the high-*T*_c_ or strongly correlated superconductors. We also emphasize that it is difficult to reconcile the strong-coupling effect with the electron–phonon coupling alone^25^. Our results establish the importance of both molecular characteristics, absent in the atom-based superconductors, involving the dynamical JT effect and the resulting renormalization of the electronic structure and electron correlation effects for both the high-*T*_c_ and the high-*H*_c2_ in the fullerides, as supported by the recent theoretical calculations^33^. This provides a new perspective on realizing robust superconductivity with high *T*_c_ and *H*_c2_ in molecular materials.

## Methods

### Sample synthesis and characterization

Fullerene superconductors Na_2_CsC_60_, K_3_C_60_, and Rb_*x*_Cs_3−*x*_C_60_ (0<*x*≦3) were synthesized by solid-vapor reaction method as described in ref. 9. The samples used here were identical to those in ref. 9. For Rb_*x*_Cs_3−*x*_C_60_ with *x*=0.5, 1, and 2, our samples correspond to Rb_0.5_Cs_2.5_C_60_ (Sample I), RbCs_2_C_60_ (Sample I), and Rb_2_CsC_60_ (Sample II) in ref. 9, respectively. The samples were characterized by synchrotron X-ray powder diffraction and magnetization measurements. The phase fraction of the fcc phase was larger than 70% and typical shielding fraction was ∼90%.

### Measurements of *H*_c2_

Contactless radiofrequency (r.f.) penetration depth measurements were performed using a proximity detector oscillator technique^13^ and a pulsed magnetic field up to 62 T in Los Alamos NHMFL. The typical resonant frequency was ∼28 MHz. The r.f. technique is highly sensitive to small changes (approximately 1–5 nm) in the r.f. penetration depth *λ*, and thus, it is an accurate method for determining *H*_c2_ of superconductors. Powder samples were compressed into pellets and sealed in thin glass capillaries with a small amount of He gas. Coils that generate and detect microwave signals are directly wound around the capillary (inset of Fig. 2a). The relative change of *λ* is proportional to the relative change of the resonating frequency *f* through the inductance of the coil, that is, Δ*f*/*f*∝Δ*λ*/*λ* (ref. 13). Upper critical field *H*_*c*2_ was determined from the field dependence of the frequency shift Δ*f* (Supplementary Fig. 1) as the point at which the slope of the r.f. signal in the superconducting state intercepts the slope of the normal state background.

### Data availability

The data that support the findings of this study are available on request from the corresponding authors (Y.K. or Y.I.).

## Additional information

**How to cite this article:** Kasahara, Y. *et al*. Upper critical field reaches 90 Tesla near the Mott transition in fulleride superconductors. *Nat. Commun.* **8,** 14467 doi: 10.1038/ncomms14467 (2017).

**Publisher's note**: Springer Nature remains neutral with regard to jurisdictional claims in published maps and institutional affiliations.

## Supplementary Material

Supplementary Information Supplementary Figures 1-2, Supplementary Table 1, Supplementary Note 1 and Supplementary References

Peer Review File
