# Supplementary material for: Upper critical field reaches 90 tesla near the Mott transition in fulleride superconductors
Source: Nat Commun. 2017 Feb 17;8:14467. doi: 10.1038/ncomms14467 (PMC5321754; doi:10.1038/ncomms14467)
Supplement: Supplementary Information — Supplementary Figures 1-2, Supplementary Table 1, Supplementary Note 1 and Supplementary References [file ncomms14467-s1.pdf]

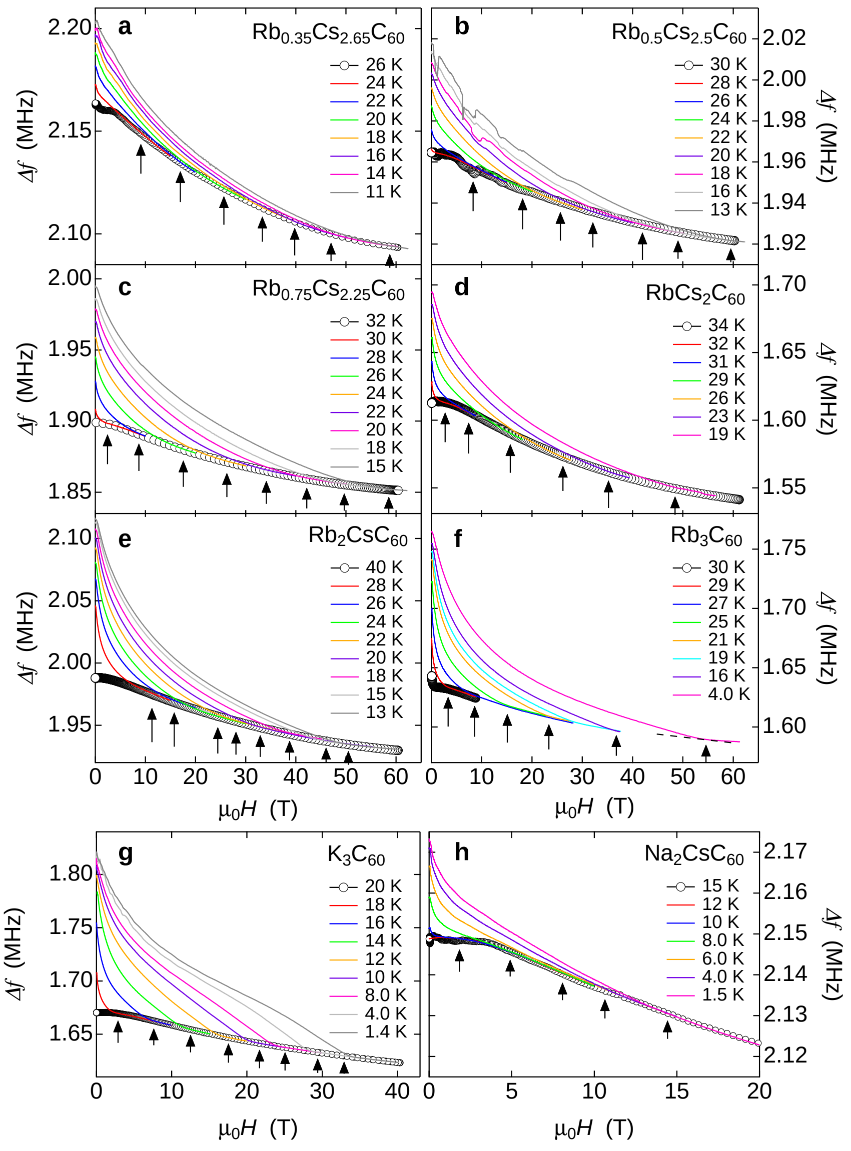

Supplementary Figure 1. **Temperature dependence of frequency shift.** Temperature dependence of frequency shift (Δf) as a function of magnetic field in $Rb_xCs_{3-x}C_{60}$, K_3C_{60}, and Na_2CsC_{60}. Open circles are Δf taken at $T > T_c$ as a normal-state background signal. The arrows indicate $H_{c2}(T)$ determined from the point deviating from the background signal. All data were taken under the down-sweep of pulsed magnetic fields. Upper critical field H_{c2} (arrows) was determined as the point at which the slope of the rf signal in the superconducting state intercepts the slope of the normal state background. For Rb_3C_{60} at 4.0 K, although the normal state background up to 60 T is missing, H_{c2} is determined as the point at which the slope of the rf signal in the superconducting state intercepts the extrapolation of the normal state signal (dashed line).

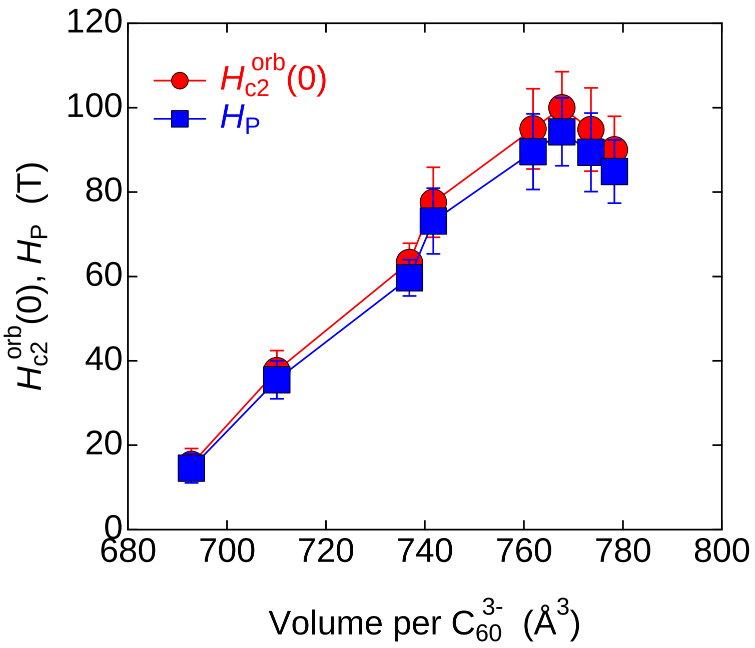

Supplementary Figure 2. **Volume dependence of orbital and Pauli limiting fields.** $H_{\rm c2}^{\rm orb}(0)$ and $H_{\rm P}$ as a function of volume per $\rm C_{60}^{3-}$ anions. $H_{\rm c2}^{\rm orb}(0)$ is estimated from the initial slope of $H_{\rm c2}(T)$ near $T_{\rm c}$. $H_{\rm P}$ is obtained from the fits to $H_{c2}(T)$ using eq. (1) in the main text. Error bars represent s.d. of the fit to $H_{c2}(T)$ curves.

Supplementary Table 1. Summary of the parameters for the upper critical fields. Here, $H_\mathrm{P}^\mathrm{BCS}[\mathrm{T}] = 1.84T_\mathrm{c}[\mathrm{K}]$.

Material	$\left\|\frac{dH_{c2}(T)}{dT}\right\|_{T_c}$ (T/K)	$H_{c2}^{orb}(0)$ (T)	ξ_{GL} (nm)	H_P^{BCS} (T)	H_P (T)	$H_{c2}(0)$ (T)
Na_2CsC_{60}	1.96	15.5	4.63	21.0	21.0	13.5
K_3C_{60}	2.87	37.7	2.95	35.0	35.5	33.0
Rb_3C_{60}	3.12	63.3	2.28	54.1	59.7	57.0
Rb_2CsC_{60}	3.58	77.6	2.06	57.8	73.1	68.0
$RbCs_2C_{60}$	4.24	95.0	1.86	59.8	89.6	85.0
$Rb_{0.75}Cs_{2.25}C_{60}$	4.62	100	1.81	57.8	94.3	88.0
$Rb_{0.5}Cs_{2.5}C_{60}$	4.67	94.8	1.86	54.1	89.4	84.0
$Rb_{0.35}Cs_{2.65}C_{60}$	4.85	90.0	1.91	49.5	84.9	77.0

Supplementary Note 1

Volume evolution of orbital and Pauli limiting fields.

Orbital limiting field $H_{c2}^{orb}(0)$ and Pauli limiting fields H_P were estimated from the measured upper critical field $H_{c2}(T)$. Throughout the analysis, we assumed that the fullerides are in the dirty limit, since $\xi_0 \sim \ell$, where ξ_0 is the superconducting coherence length and ℓ is the mean free path, which has been reported by high frequency resistivity measurements for K_3C_{60} [1]. First, $H_{c2}^{orb}(0)$ has been estimated from the initial slope of $H_{c2}(T)$ near T_c through the relation of $H_{c2}^{orb}(0) = 0.69T_c|dH_{c2}/dT|_{T=T_c}$. The Ginzburg-Landau coherence length ξ_{GL} is estimated from $H_{c2}^{orb}(0) = \Phi_0/2\pi\xi_{GL}^2$ (Supplementary Table S1). As seen in Fig. 3c of the main text, the normalized $H_{c2}(T)$, $(H_{c2}(T)/T_c)/|dH_{c2}/dT|_{T_c}$, collapse into a single curve except for Na_2CsC_{60}. This is presumably because the structure is distinct between Na_2CsC_{60} (simple cubic) and the other compositions (face centered cubic). Therefore, we merged $(H_{c2}(T)/T_c)/|dH_{c2}/dT|_{T_c}$ data for $Rb_xCs_{3-x}C_{60}$ and K_3C_{60}, and then, fitted the merged data by eq. (1) in the main text, to derive the set of fitting parameters (α, λ_{so}), where $\alpha = \sqrt{2}H_{c2}^{orb}(0)/H_P$ and λ_{so} is the spin-orbit scattering rate. $(\alpha, \lambda_{so}) = (1.5, 4.4)$ is obtained for $Rb_xCs_{3-x}C_{60}$ and K_3C_{60}, and $(\alpha, \lambda_{so}) = (1.0, 2.8)$ for Na_2CsC_{60}. H_P is estimated from $H_P = \sqrt{2}\Delta_0/g\mu_B$ (g is g-factor of electron spins and μ_B is the Bohr magneton), assuming that $g = 2$, which has been reported for several A_3C_{60} compounds [2]. The parameters related to the above analysis are summarized in Supplementary Table 1, and the volume dependence of $H_{c2}(0)$ and H_P is shown in Supplementary Figure 2.

[1] Klein, O., Grüner, G., Huang, S.-M., Wiley, J. B. & Kaner, R. B. Electrical resistivity of K_3C_{60}. *Phys. Rev. B* **46**, 11247-11249 (1992).

[2] Tanigaki, K. *et al.* Alkali effects on the electronic states of K_3C_{60} and Rb_3C_{60}. *Chem. Phys. Lett.* **240**, 627-632 (1995).
